# Do explainable AI (XAI) methods improve the acceptance of AI in clinical practice? An evaluation of XAI methods on Gleason grading

**DOI:** 10.1002/2056-4538.70023

**Published:** 2025-03-13

**Authors:** Robin Manz, Jonas Bäcker, Samantha Cramer, Philip Meyer, Dominik Müller, Anna Muzalyova, Lukas Rentschler, Christoph Wengenmayr, Ludwig Christian Hinske, Ralf Huss, Johannes Raffler, Iñaki Soto‐Rey

**Affiliations:** ^1^ Digital Medicine University Hospital of Augsburg Augsburg Germany; ^2^ IT‐Infrastructure for Translational Medical Research University of Augsburg Augsburg Germany; ^3^ Institute for Pathology and Molecular Diagnostics University Hospital of Augsburg Augsburg Germany; ^4^ BioM Biotech Cluster Development GmbH Planegg Germany; ^5^ Bavarian Cancer Research Center (BZKF) Augsburg Germany

**Keywords:** explainable artificial intelligence, artificial intelligence, evaluation, Gleason score, prostate carcinoma, pathology, clinical decision support

## Abstract

This work aimed to evaluate both the usefulness and user acceptance of five gradient‐based explainable artificial intelligence (XAI) methods in the use case of a prostate carcinoma clinical decision support system environment. In addition, we aimed to determine whether XAI helps to increase the acceptance of artificial intelligence (AI) and recommend a particular method for this use case. The evaluation was conducted on a tool developed in‐house with different visualization approaches to the AI‐generated Gleason grade and the corresponding XAI explanations on top of the original slide. The study was a heuristic evaluation of five XAI methods. The participants were 15 pathologists from the University Hospital of Augsburg with a wide range of experience in Gleason grading and AI. The evaluation consisted of a user information form, short questionnaires on each XAI method, a ranking of the methods, and a general questionnaire to evaluate the performance and usefulness of the AI. There were significant differences between the ratings of the methods, with Grad‐CAM++ performing best. Both AI decision support and XAI explanations were seen as helpful by the majority of participants. In conclusion, our pilot study suggests that the evaluated XAI methods can indeed improve the usefulness and acceptance of AI. The results obtained are a good indicator, but further studies involving larger sample sizes are warranted to draw more definitive conclusions.

## Introduction

Algorithmic classification of whole slide images using deep‐learning systems in pathology has been shown to be at least on par with clinical experts in certain use cases regarding their accuracy and demonstrates potential for assisting in clinical decisions [1, 2, 3, 4]. In clinical practice, however, very few artificial intelligence (AI)‐driven clinical decision support systems (CDSS) have been established to date [5].

One reason for this is that deep‐learning methods such as neural networks often appear as ‘black boxes’ to the end user, which creates a feeling of discomfort in trusting AI‐generated decisions. One major influence is the lack of a basic understanding of how AI works, its limitations, and its informational value [6]. Additionally, there are legal requirements in several countries (e.g., the Medical Device Regulations of the European Union [7]) that assistance systems must transparently explain to the user why a procedure is proposed.

If we want to harness the benefits of AI, we need systems that provide insights into the underlying arguments for AI‐based decision‐making. The research area of explainable AI (XAI) aims to unveil the ‘black box’ of neuronal networks with different approaches [8]. Initial XAI approaches were primarily meant to be used by developers of AI models, mainly for validation and debugging. However, recent efforts also aim to help the end users understand the inner mechanisms of the models [9, 10, 11].

The adoption of XAI into medical care is still in its early stages. Studies evaluating different explanation types have been performed before [12]. However, to our knowledge, very few XAI methods have been evaluated regarding their usefulness from the perspective of the end users, and no established frameworks for this kind of evaluation exist.

For image‐based classification systems such as those used for digitized histopathological slides (whole slide images), XAI methods that highlight the areas relevant to the decision are particularly suitable to provide additional insights [13].

In our EKIPRO (Explainable Artificial Intelligence for Prostate Carcinoma) project, we address this lack of knowledge by systematically evaluating the most common XAI visualizations on the use case of Gleason grading. We chose prostate carcinoma, a well‐understood, broadly investigated, and very prevalent form of cancer, as our use case [14]. The Gleason Score [15] is a widely used method for risk assessment and therapy decision‐making for prostate carcinoma [16, 17]. Determination of the Gleason Score is mainly based on pattern recognition, which is known to be replicable by neural networks. Thus, reliant and accurate systems, often outperforming individual physicians, have been demonstrated and could be used to enhance decision‐making [1, 2, 3, 4]. However, the lack of interpretability of AI models is a major issue that hampers trust and may impact user satisfaction in CDSS. This issue could be addressed by integrating XAI methods.

In this study, we aimed to address three main questions. First, which gradient‐based XAI method is best for the use case of prostate carcinoma? Second, are XAI methods helpful, transparent, and understandable for pathologists regarding decision‐making? And lastly, does XAI raise the acceptance of AI in clinical practice?

To this end, we collected our own annotated dataset, developed a deep learning model to predict Gleason grades, and created an evaluation tool within a clinical decision support (CDS) setting.

## Background

### Deep learning and convolutional neural networks

Deep learning, a subset of machine learning, has transformed various fields by enabling machines to learn from data representations [18]. Central to this paradigm are convolutional neural networks (CNNs), which excel in tasks like image classification and object detection [19]. CNNs find applications in computer vision and especially in medical image analysis, owing to their ability to learn complex representations from diverse datasets [20]. Their versatility and performance make them indispensable in modern medical AI research.

### Explainable artificial intelligence

XAI is a research area that aims to make machine learning systems more understandable and interpretable. The XAI systems can benefit the end users by providing additional arguments for their decisions, as well as the developers, as XAI can be used to ensure the system performs as intended [21].

In the medical context, XAI creates an interface between the clinician and the AI by giving more context about the underlying AI model in a comprehensive way [21, 22].

XAI methods can be categorized according to several attributes, such as explanation, location, or modality. The latter can be text, concepts, examples (e.g., counterfactuals) or feature attribution [23].

For medical image analysis with neural networks, post hoc gradient‐based feature attribution methods are broadly used. They provide visual explanations (images) by highlighting the important individual pixels or larger areas of the input image that could be influencing the AI's decision. The XAI output is usually displayed as a heatmap overlaying the input image.

### Usability in healthcare

Usability is defined by the ISO 9241‐11 as ‘the extent to which a product can be used by specified users to achieve specified goals with effectiveness, efficiency, and satisfaction in a specified context of use’ [24]. There are several methods to measure or evaluate usability [25, 26, 27]. These methods generally involve user‐based testing and expert‐based inspections [28]. Methods such as ‘think aloud’ can provide additional information by capturing the impressions of the end users while they are testing the designed product or system [29].

The usability of implemented systems and tools becomes crucial in high‐stake decision fields such as healthcare [30, 31, 32]. The healthcare workers must be able to use the information systems and tools with as few errors as possible to ensure the patients safety [27].

Considering the impact and high performance of AI, systems implementing AI could improve the clinical routine immensely. However, these systems are rarely used due to the lack of explainability and usability.

### Prostate cancer

Prostate cancer is one of the most diagnosed malignancies in men worldwide and accounts for 3.8% of all cancer‐related deaths [14]. The vast majority of prostate cancer is acinar adenocarcinoma [33]. Diagnosis of prostate cancer is usually made after clinical suspicion and confirmed by transrectal ultrasound‐guided core needle biopsies, with subsequent histopathological assessment by a pathologist [34]. The international standard for histopathological grading in prostate cancer is the Gleason grading [16, 17]. Established in 1966, the Gleason grading system classifies prostate adenocarcinomas by their growth patterns. While the original grading described five different growth patterns assigned numbers 1–5, patterns 1 and 2, accounting for the most well‐differentiated tumors, have practically disappeared from clinical practice, leaving the commonly used grades 3, 4, and 5 (supplementary material, Figure S1).

Despite the long‐established classification based on Gleason grading, one of the main problems of classifying prostate cancer is the considerable interobserver variability, ranging mostly in the fair to moderate agreement range [35, 36, 37, 38]. Although in recent years, it has been shown that interobserver variability can be reduced by using artificial intelligence‐based support tools [39].

## Study context

### Organizational setting

The study was conducted at the University Hospital of Augsburg (UKA), a tertiary healthcare facility for the region Augsburg–Swabia. Members of UKA's pathology department acquired and annotated the dataset. The development of the AI pipeline and evaluation software was done both at the Institute for Digital Medicine (IDM) of UKA and the chair of IT infrastructures for translational medical research of the University of Augsburg. All participants who took part in the evaluation were physicians from the UKA's pathology department.

### System details and system in use

We chose a representative sample of the most popular XAI methods for CNNs. We also considered the constraints of scalability for computing costs regarding the size of the target images.

The selected methods were the gradient‐based methods as follows:

Grad‐CAM (GC) or gradient‐weighted class activation mapping is a popular technique that highlights the regions of an image that are most important for a specific class prediction. It uses the gradients of the target class with respect to the last convolutional feature maps to produce a heat map. The resulting heat map indicates the broad region that contributed most to the prediction. This method outperformed all other available methods in localization, pointing, and faithfulness by the time of publication [40].

Grad‐CAM++ (GC++) is a method building on the previous method, GC. It incorporates additional features such as the weighted combination of the positive partial derivatives of the last convolutional layer. This results in an improved result compared to GC++ in most scenarios. Specifically, it improves the detection of multiple occurrences of the same class and the localization of objects in the image [41].

Integrated gradients (IGs) are mathematical methods based on mathematical axioms of sensitivity and implementation invariance, which calculate the gradient of the input image in relation to a baseline image (usually completely black). By integrating the gradients, the contribution of each pixel to the final prediction can be measured. This results in a very fine scattered image with details at the pixel level since it does not include relations to neighboring pixels. While this method has a solid theoretical foundation, the resulting heat maps are often granular and hard to interpret [42].

Guided backpropagation (GB) works similarly to the common backpropagation algorithm [43] but adds a guidance signal from higher layers. Depending on the input image, the heat map highlights many small features such as edges and specific shapes relevant for the prediction. This represents a visualization of features learned by higher layers in the convolutional network and can be used for the interpretation of the detected class [44].

Saliency maps (SM) refer to two different concepts. While one describes a whole subset of AI explainability, it was also introduced as the name of a concrete XAI method to which we are referring This method computes the relevance of areas on the sample (like GC), but instead selects the most significant feature for each channel. The result is a SM of the given image that is discriminative to the predicted class [45].

**Figure 1:**
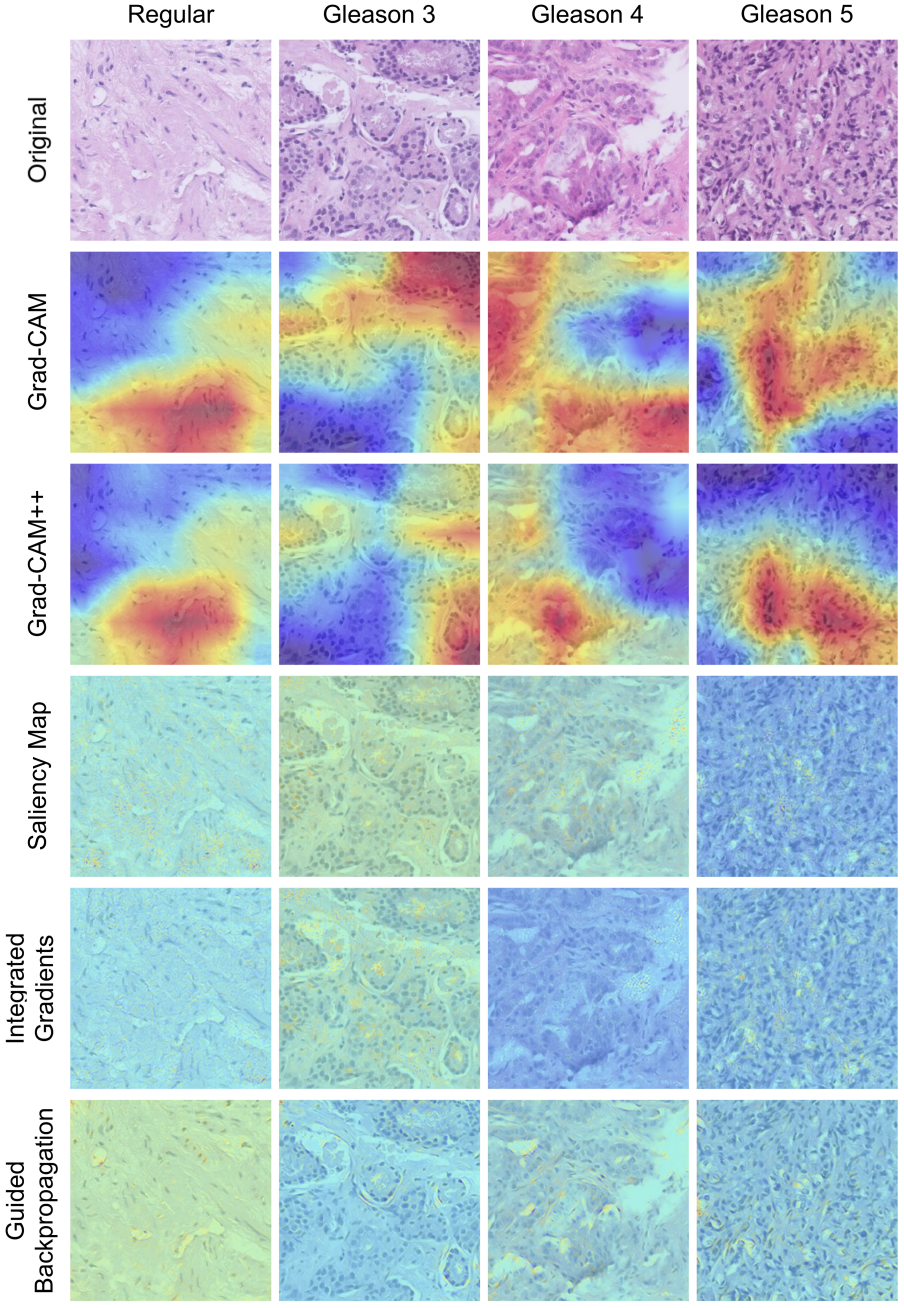
Examples of results produced by the five XAI methods examined in this study, displayed as color gradients overlaying the original image of the sample.

In simple terms, GC and GC++ highlight broader regions of the image, whereas IG, GB, and SM work on a finer detailed pixel level (Figure 1).

## Materials and methods

### Usability evaluation study design

We conducted a questionnaire‐based, cross‐sectional study at the UKA in a simulated CDS setting using a self‐developed evaluation tool. The study was conducted at the facilities of the UKA's Pathology department under the supervision of the IDM study team. All 15 pathologists from the Pathology department of the UKA participated in this study. The evaluation sessions were conducted in small groups with one to five participants, each with one or two supervisors.

The study was approved by the ethics committee of the Ludwig‐Maximilians‐University (LMU) Munich, Germany (‘Ethikkommission der Medizinischen Fakultät der LMU’), approval number 21_0543.

### AI model and XAI

Using the laboratory information system of the Institute for Pathology and Molecular Diagnostics at the UKA, we identified 325 prostate cancer cases from 2019 to 2021. A total of 1,202 slides with H&E‐stained histological sections of prostate needle biopsies correspond to these cases. A total of 620 of these slides have been digitized using a Phillips UltraFast Scanner at ×40 magnification; 369 of the digitized slides (corresponding to 93 cases) were partially annotated in QuPath (V.0.3.0) [46] by a junior pathologist under the supervision of a senior pathologist (supplementary material, Figure S2). For the annotation, image regions were marked that either represent regular tissue, carcinoma tissue with Gleason grades of 3, 4, or 5, artefacts such as air pockets or slide contamination, and questionable regions without definitive classification.

For AI pipeline development and XAI generation, we utilized the open‐source AUCMEDI framework [47]. The 369 digitized and annotated slides were split into a training and validation dataset (295) and a testing dataset (74). The slides were preprocessed by dividing them into tiles of 1,024 × 1,024 pixels, corresponding to 256 × 256 μm on the glass slide. A ResNeXt101 deep neural network was employed to classify tiles into categories for Gleason grading, achieving strong predictive capabilities with a macro‐averaged F1‐score of 0.756, area under the curve (AUC) of 0.989, and an accuracy of 0.982 on a hold‐out testing set (supplementary material, Figures S3 and S4).

For the XAI module, Gleason gradings were inferred for all tiles using the AI model. Based on these predictions, two sets of XAI overlays were generated. The first set included one overlay per slide for each XAI method. It visualizes the average attention over all classes which approximates toward highlighting the dominant predicted classes model (‘general attention overlay’). The second set of overlays was generated per slide for each class and for each XAI method, displaying the AI attention based on the assumption the corresponding class was predicted (‘class‐based overlay’). For a more intuitive visualization, class‐based overlays for tiles with a predicted class probability below a threshold of 0.3 were skipped. A detailed explanation of the coloring for each of the five methods, including the representation of low and high agreement between them, can be found in supplementary material, Figure S5.

### Usability evaluation and data collection

#### Questionnaire design

The questions were designed together with clinical and usability experts to capture the perceived usefulness of the methods using a five‐point Likert scale. The evaluation consisted of two online questionnaires and a ranking.

The first questionnaire collects demographic information about the users, such as expertise in pathology and Gleason grading, age group, as well as interest and experience in AI. This information is used to analyze correlations, for example, between experience and openness toward AI (supplementary material, File S1).

The second questionnaire covers the perceived performance of the XAI methods and the AI in general. It is composed of seven sections and is completed once per slide. The first five sections correspond to each XAI method. They are identical and include five‐point Likert scale questions (1 = strongly disagreed to 5 = strongly agreed), one binary choice, and a free‐text area. The order of these five XAI method sections was randomized to prevent Question order bias. With these questions, we aim to gather comparative data between methods and their performance in a CDS setting. This is followed by a ranking where the evaluator is asked to classify the five different XAI methods with a prioritization from the most useful to the least useful. The last section of the questionnaire includes eight Likert scale questions regarding the general perception of the AI classification (Gleason grading) and its usefulness in a CDS context. The aim of this section was to identify general trends in AI and its application in pathology (supplementary material, File S2).

#### Evaluation tool

**Figure 2:**
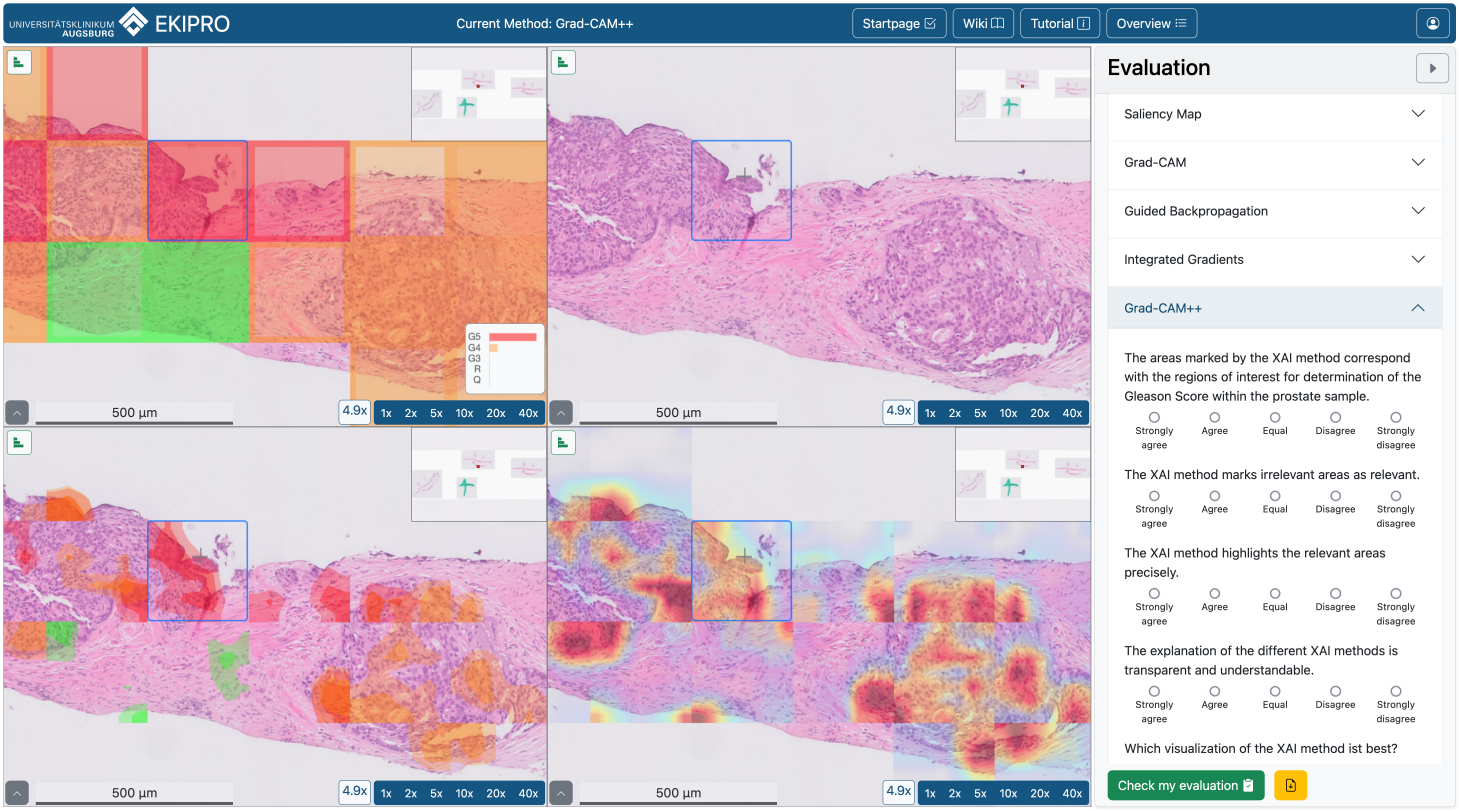
Screenshot of the EKIPRO tool used for the evaluation. Top left: Gleason overlay (green, regular; yellow, Gleason 3; orange, Gleason 4; red, Gleason 5). Top mid: sample with no overlays. Bottom left: class‐based overlay. Bottom mid: general attention overlay. Right: Questionnaire.

Due to the lack of existing tools for application‐grounded evaluations that allowed for the complex visualization required, we implemented our own. With the help of user‐centered design methods, we designed an intuitive user interface to align with pathologists' requirements and expectations. The design and development of the tool were conducted at the IDM in close collaboration with the Pathology department of the UKA. We opted for a web application to ensure easy access from various devices without complex installations, promoting widespread usability. For seamless integration, we used Docker containers, which facilitate deployment and scalability (Figure 2 and supplementary material, Figure S5).

The tool includes the following functionality: (1) four synchronized viewers that always display the base image; (2) an adaptable overlay configuration to display the tile‐based XAI heatmaps as well as the tile‐based Gleason classification on top; (3) a bar chart depicting the certainty of each classification; (4) an adjustable colormap selection to colorize the heatmaps in different color schemes; (5) a dynamic form to display the questionnaire and collect the data in parallel to the viewer; (6) a basic user management system to keep track of the participants; (7) a pre‐evaluation form to collect meta‐data; and (8) a tutorial and Wiki page to provide additional clarification and an extended introduction to the topic.

The tool was programmed using Django. We used a sqlite3 database, complemented by a HTML and jQuery frontend, using the Bootstrap framework. For visualizing both slides and overlays, we developed an extension for the Openseadragon Framework.

A demo version of our tool is available at https://ekipro.idm.uk-augsburg.science.

#### The evaluation

All participants were informed about the scientific background and the goals of the study in an introduction session, where key AI/XAI concepts (e.g., explainability and interpretability as defined in Marcinkevičs and Vogt [48]) as well as a short tutorial for using our evaluation tool were presented.

The evaluation sessions took place in groups of one to five participants for 60–90 min. In each session, one or two researchers were present as supervisors to provide clarification on topics like AI and XAI and the handling of the tool. The sessions were also held using the ‘think aloud’ method to gather additional information. We selected 10 slides (corresponding to 10 distinct cases) of our dataset for the evaluation where the Gleason grading of the AI was generally accurate. These 10 slides were neither used for the training nor for the validation of the AI model. The order of the slides was shuffled for each participant. The participants were asked to evaluate as many samples as possible within their timeframe. Data was collected between 19 April 2023 and 7 June 2023.

### Data analysis

For data management and statistical analysis, we used SPSS version 28.0. For the descriptive analysis and chart generation, we used Microsoft Excel 2019. The categorical variables such as age category, experience with AI, XAI systems, and Gleason Score are presented as absolute frequencies and percentages. For descriptive analysis, we calculated means and standard deviations of the Likert scale questions. To screen for significant differences, we used Kruskal–Wallis tests. We analyzed correlations between interval‐scaled (age, binned experience as pathologist) and/or ordinal‐scaled (Likert‐type questions, AI and Gleason experience) variables with Spearman's rho coefficients. Dependencies between categorical variables as well as the differences in ranking of XAI methods were tested with the help of the chi‐squared test. We report Bonferroni‐corrected *p* values where applicable at a nominal significance level of 0.05. The free‐text answers were analyzed and classified as positive or negative comments toward the XAI method, generating a positive and negative score on each XAI method and overlay presentation.

## Results

### Study findings and outcome data

All of the participants (15) completed the evaluation of at least one slide, resulting in a total of 44 completed evaluations on 10 slides.

#### Demographics

Most of the participants (9/15) were between 26 and 35 years old and had 1–5 years of experience in pathology. All but one of the participants had previous experience with the Gleason Scoring system and used it at least once per month. The knowledge of the Gleason grading system was rated predominantly ‘good’ or better (9/15). Slightly less than half of the participants had no experience with AI in pathology before (7/15). All participants showed at least some interest in AI solutions for pathology. Eight of the participants stated they had at least heard about XAI (Table 1).

**Table 1:** Demographics

Variable	*N*	%
Gleason Score experience		
Very low	0	0
Low	3	20
Moderate	3	20
Good	4	26.7
A lot	5	33.3
Gleason Score frequency in daily work		
Never	0	0
Yearly	1	6.7
Monthly	7	46.7
Weekly	6	40
Daily	1	6.7
Years of experience in pathology		
1–5	9	60
6–10	2	13.3
11–15	1	6.7
16–20	3	20
21+	1	6.7
Age of participants		
<25	0	
26–35	9	60
36–45	2	13.3
46–55	1	6.7
>55	3	20
Interest in AI		
No interest in AI	0	
Some interest	6	40
Moderate interest	5	33.3
Interested	3	20
Very interested	1	6.7
Experience with AI		
No	8	53.3
Very low	0	0
Low	1	6.7
Moderate	3	20
Good	3	20
A lot	0	0

#### XAI evaluation

**Figure 3:**
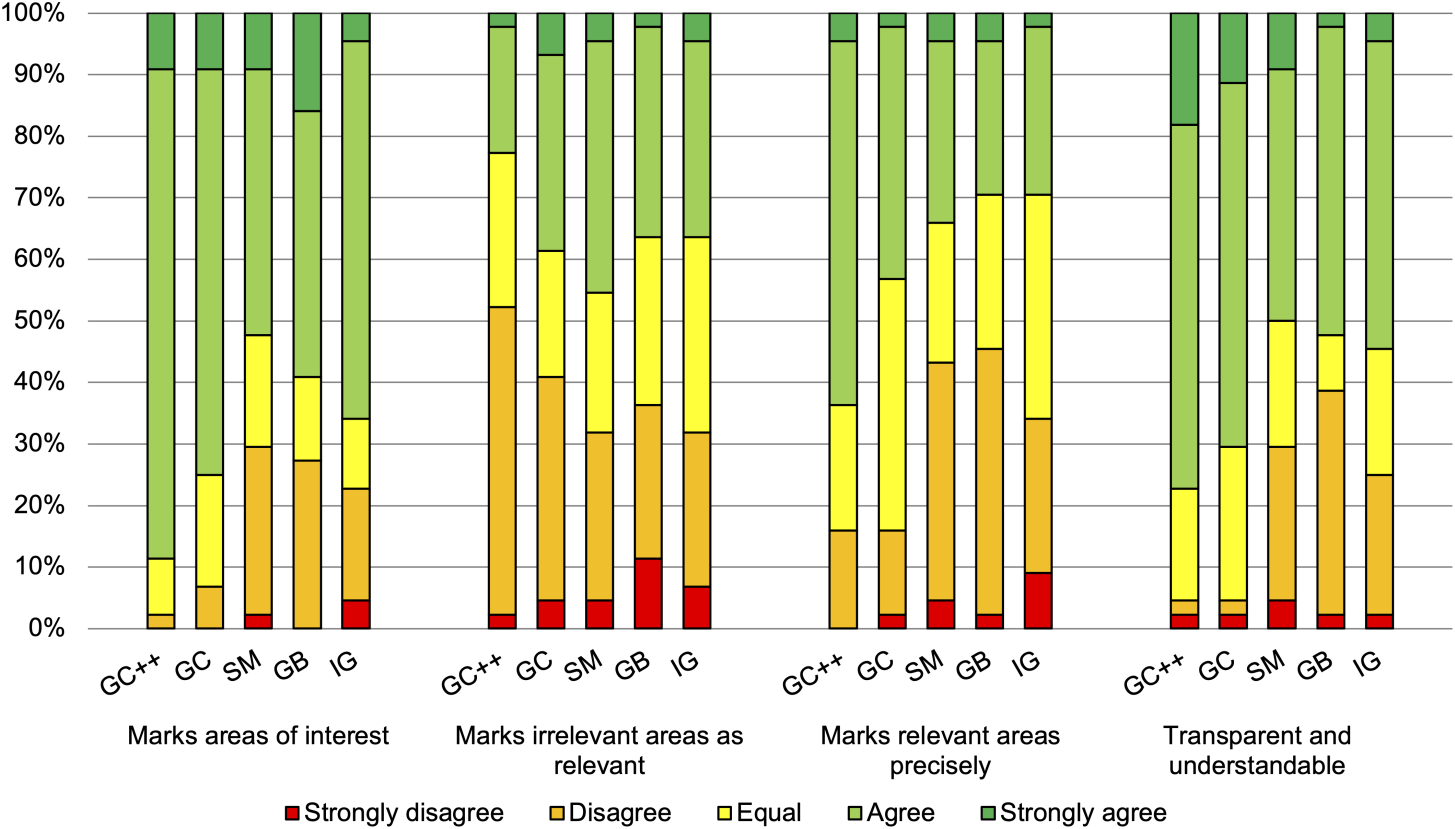
Agreement Likert‐scale answers per XAI method.

In total, we evaluated five XAI methods based on four questions (Figure 3). For three of these four questions, we identified significant differences (*p* values ≤0.05, adjusted for multiple testing) in the Likert‐scaled answers given by the participants. In all cases, GC++ outperformed the other methods.

On the question ‘Do the areas marked correspond with the regions of interest’, GC++ scored significantly better (3.95 ± 0.53) than SMs (3.30 ± 1.05, *p* = 0.013). Furthermore, regarding the question ‘Does the method mark the areas of interest precisely’, GC++ was rated significantly better (3.52 ± 0.82) in comparison to GB (2.86 ± 0.98, *p* < 0.01), IGs (2.89 ± 0.99, *p* = 0.02), and SMs (2.91 ± 1.03, *p* = 0.021). And finally, regarding the question ‘Is the explanation transparent and understandable’, GC++ was rated significantly better (3.89 ± 0.81) compared to SMs (3.25 ± 1.08, *p* < 0.025) and GB (3.14 ± 1.03, *p* < 0.01).

The free‐text answers, categorized into positive (+) comments and negative (−) comments, reflect the results from the statistical analysis. GC++ was most favored (6+, 4−), followed by GC (8+, 8−), guided backpropagation (3+, 5−), SMs (3−), and IGs (5−) (supplementary material, File S3).

Regarding the visualization, the class‐based overlay was preferred over the general attention overlay for all XAI methods but GB, where both visualizations were equally rated (50%). In the case of GC++ and GC, the preference for the class‐based overlay was prevalent with 31 and 33 out of 44 total answers, respectively (supplementary material, Table S1).

Additionally, the class‐based overlay received six positive comments, whereas the general attention overlay received three positive and four negative comments.

#### Ranking

**Figure 4:**
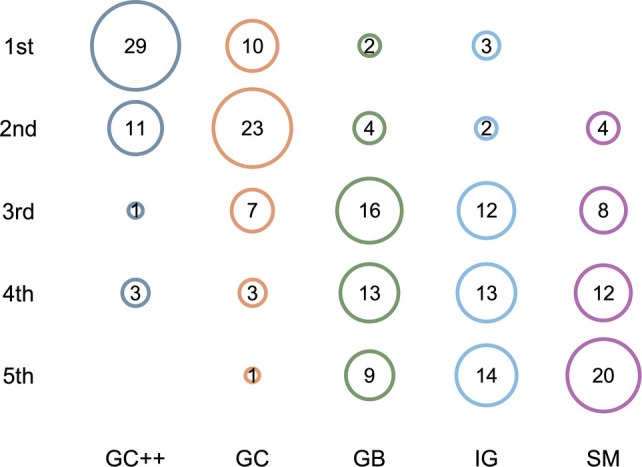
Bubble chart of the ranking of XAI methods.

The results of the XAI methods ranking showed that GC++ was placed significantly more frequently in position 1 (65.9%, *p* < 0.01), followed by GC (52.3%, *p* < 0.01). IGs was ranked third (36.40%) and GB fourth (29.5%), both showing no significance. SMs was rated worst since it was significantly more frequently placed in rank 5 (45.5%, *p* < 0.01) when compared to the other methods (Figure 4).

#### General evaluation

**Figure 5:**
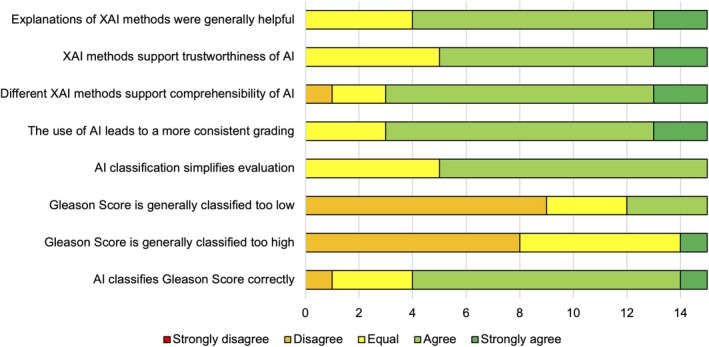
General evaluation results of the five‐point Likert scale answers.

When asked about the general usefulness of AI and XAI, 29 out of the 44 answers agreed that the XAI methods were helpful. Twenty‐seven of the answers agreed that the XAI improved trustworthiness. Thirty‐six answers agreed that the XAI methods supported the comprehensibility of the AI and verified that the use of AI leads to a more consistent grading. In 30 answers, the AI was agreed upon to simplify the evaluation process. Twenty‐nine answers stated that the scores were not rated as being too low or too high (Figure 5).

#### Correlations

The frequency of examining prostate slides in day‐to‐day work was positively correlated with both the trustworthiness (*r* = 0.63, *p* < 0.011) and the helpfulness (*r* = 0.57, *p* < 0.027) of AI classifications. Furthermore, there is a non‐significant indication that the frequency of prostate examinations positively correlates with the agreement on XAI supporting the comprehensibility of AI systems (*r* = 0.483, *p* < 0.068).

### Unexpected observations

The participants were highly concentrated on task completion and were not able to provide a thorough description of their thoughts for a fully successful usage of the Think‐Aloud method. However, we determined that the differentiation between AI classification and the corresponding XAI explanation was hard to comprehend. Furthermore, the colormap from the general overlay ranging from blue to red caused confusion. Red areas were usually depicted as malignant and collided with the XAI, using red to mark the respective areas of highest interest.

## Discussion

### Answer to study questions

The results of our evaluation show a clear preference for the GC++ method in a simulation of a CDSS. GC++ was ranked best in marking the regions of interest, highlighting the relevant areas, and was rated as the most transparent and understandable method. In contrast, methods producing more granular visualizations such as SMs proved to be incomprehensible and their results difficult to understand. The class‐based overlay to represent the XAI heatmap was found to be the most comprehensible representation. Integrating the Gleason grading color scheme made the overlay easier to interpret. In addition, by reducing complexity with discrete transparency levels and a cut‐off threshold for unimportant areas, the overlay became more readable and easier to interpret.

XAI in general was found to be helpful by participants in their decision‐making. It increased the trustworthiness and understandability of the AI prediction and indicated an increased acceptance of the AI.

### Results in relation to other studies

Computational pathology is a rapidly evolving field that holds immense importance in modern healthcare. The role of the pathologist as primary users of digital pathology systems has been thoroughly investigated [49]. However, to the best of our knowledge, application‐grounded evaluations requiring experts [23] of XAI on image data have only been conducted in rare instances and for very specific use cases. Examples of such use cases are [50, 51]. However, these use cases did not use the same assortment of methods we tested. To our knowledge, there has not been an application‐grounded evaluation done using the same methods and use case; thus, Gleason grading, as us, rendered results ineffective.

Nevertheless, an assortment of papers has been published documenting research within the field of digital pathology and displaying the usefulness of XAI within. Most research, including an evaluation of the presented XAI methods, uses gradient‐based methods displaying the XAI results visually to the user [12, 52, 53, 54, 55, 56]. This visual presentation has been found to be preferred by pathologists as it mirrors their way of thinking, allowing an easy implementation into their workflow [12]. This finding supports our choice of XAI methods.

Additional types of XAI which are commonly used within digital pathology are counterfactuals, presenting different possible model outcomes if certain features were not present [12] and model agnostic methods [57, 58], the benefit of which is their universality concerning the underlying AI system.

Similarly to other studies and reviews [51, 53, 59, 60] we have also experienced a lack of existing evaluation methods and standard procedures to determine the performance of the methods within an application‐grounded evaluation.

Since XAI research for medical use is still in its infancy, we intend to initiate more research on the topic and facilitate user‐driven evaluations.

### Meaning and generalizability of the study

We have demonstrated that XAI can enhance the experience of physicians using AI tools and provide more context for the decision‐making process and thus can build more trust in the CDSS. Especially the GC++ method showed a promising acceptance and performance, which makes it conceivable for further use in productive systems. Further work on education about the potentials and limitations of AI must be performed for a successful adoption in clinical practice. Furthermore, the way the heatmaps are visualized and interpreted impacts the usability and requires more research. Our way of representation with the class‐based overlay proved to be effective, but several alternative possibilities can be explored.

Our study mainly targets the use case of Gleason grading. However, the evaluation plan and the developed tool can be reused for further evaluations on AI and XAI.

### Unanswered questions

Due to the scope of this study, we did not investigate further XAI configuration parameters such as colormaps, composition methods, cut‐off thresholds, and transparencies. Also, other popular saliency‐based XAI methods such as DeconvNets, DeepTaylor, or SmoothGrad are yet to be evaluated. The value of XAI in identifying anomalies in AI classifications, particularly in edge cases and other challenging scenarios, could yield deeper insights into the effectiveness of different XAI representations in guiding pathologists toward detecting misclassifications and merits further investigation.

### Strengths and weaknesses of the study

Our study was conducted using our own AI pipeline named ‘DeepGleason’ on a manually annotated in‐house dataset [61]. We were able to recreate a highly performant model from a much smaller dataset than comparable models like Nagpal *et al* [1]. Our evaluation was conducted by physicians with experience in Gleason grading. Thus, the participants are potential users of similar XAI‐augmented CDSS.

Furthermore, a limitation of our XAI heatmaps is the tile‐wise computation origin and the lack of overlapping tile predictions, which could reduce the stitching effects observed in gradient‐based heatmaps (e.g., visible in Figure 2). Utilizing overlapping tile prediction could improve the consistency of heatmaps by averaging tile‐wise gradient maps, thereby providing stronger evidence for high‐impact regions in model decision‐making and reducing stitching effects.

In order to keep the complexity and computation cost within the scope of the project, we limited our XAI methods to five gradient‐based methods. More methods should be included in a future evaluation.

The evaluation included 15 pathologists and 44 evaluation cases. The number of completed evaluations varied between participants from one to five. A future multi‐centric evaluation would enhance the number of participants and the heterogeneity of the results.

When designing the evaluation, we identified a lack of literature and fitting guidelines concerning the usability evaluation of XAI methods. Thus, we developed the evaluation questionnaires to the best of our knowledge. Introducing standard XAI evaluation guidelines such as the ones suggested by Jin *et al* [59] would improve reproducibility and results comparison in further studies.

The participants were briefed only shortly about AI and XAI before the evaluation and have limited experience on the topic. With more education in this subject area, these evaluations would be easier and faster. During the evaluation, we noticed that in the case of the GC XAI method the general attention overlay was improperly represented. There, the overlay highlighted the inverse attention regions, resulting in a reduced direct visual comparability with other general attention heatmaps of the other XAI methods. However, GC still performed second best overall, suggesting a clear preference for XAI methods that highlight wider regions.

## Conclusion

Our study demonstrates the potential benefits of XAI, providing visual explanations of AI results and enhancing trust in AI. The pathologists evaluating the XAI methods preferred GC++ in a simulated CDS setting for Gleason grading of cancer tissue slide scans. During the evaluation, we perceived a lack of information regarding AI‐based systems and especially regarding XAI methods. The results of the evaluation identified certain limitations in some of the analyzed XAI methods as well as a need for increased education on the rapidly evolving fields of AI and XAI. Furthermore, the inclusion of end users (in our study primarily pathologists) must be considered when developing AI‐ and XAI‐based systems.

The developed evaluation framework, including the conceived methods and the developed tool, should be reused in further multi‐centric AI and XAI evaluations.

## Author contributions statement

RM led to the development of the evaluation tool, conducted the evaluations and drafted the paper. JB contributed to the development of the evaluation tool, conducted the evaluations and helped draft the manuscript. SC contributed to the literature research, planned and conducted the evaluations and helped draft the manuscript. PM and DM developed the AI model, integrated the XAI modules in the evaluation tool and helped draft the manuscript. AM led the statistical analysis of the data and helped draft the manuscript. LR annotated the histopathological images, provided feedback about the evaluation tool and helped draft the manuscript. CW extracted and integrated the images, contributed to the development of the evaluation tool and helped draft the manuscript. LCH and RH contributed to the study planning, provided feedback about the evaluation process and tool and helped draft the manuscript. JR and IS‐R led the EKIPRO project, planned and coordinated the development and evaluation of the tools and helped draft the manuscript. All authors reviewed and approved the final version of the manuscript.

## Supporting information


**Figure S1.** Visualization of tile classification from prostate carcinoma histological sections by the deployed deep neural network (AI) model
**Figure S2.** Annotations form a pathologist at the training dataset for Gleason grading
**Figure S3.** Area under the receiver operating characteristic curve (AUROC) of the AI pipeline
**Figure S4.** Confusion matrix of the AI pipeline
**Figure S5.** Screenshots taken from the EKIPRO evaluation tool, one for each XAI method, evaluated by the test group
**Table S1.** Preference of class‐based overlay per XAI method

**File S1.** User demographic questionnaire

**File S2.** User testing and evaluation questionnaires

**File S3.** Tester feedback on the evaluated XAI methods

## Data Availability

Study data can be made available upon reasonable request, subject to data privacy and confidentiality regulations.
