# Supplementary material for: Do explainable AI (XAI) methods improve the acceptance of AI in clinical practice? An evaluation of XAI methods on Gleason grading
Source: J Pathol Clin Res. 2025 Mar 13;11(2):e70023. doi: 10.1002/2056-4538.70023 (PMC11904816; doi:10.1002/2056-4538.70023)
Supplement: Supplementary file 1 — Figure S1. Visualization of tile classification from prostate carcinoma histological sections by the deployed deep neural network (AI) model Figure S2. Annotations form a pathologist at the training dataset for Gleason grading Figure S3. Area under the receiver operating characteristic curve (AUROC) of the AI pipeline Figure S4. Confusion matrix of the AI pipeline Figure S5. Screenshots taken from the EKIPRO evaluation tool, one for each XAI method, evaluated by the test group Table S1. Preference of class‐based overlay per XAI method [file CJP2-11-e70023-s001.pdf]

Do explainable AI (XAI) methods improve the acceptance of AI in clinical practice? An evaluation of XAI methods on Gleason grading

R Manz *et al.*, *J Pathol Clin Res,* https://doi.org/10.1002/2056-4538.70023

Supplementary Figures S1–S5
Supplementary Table S1

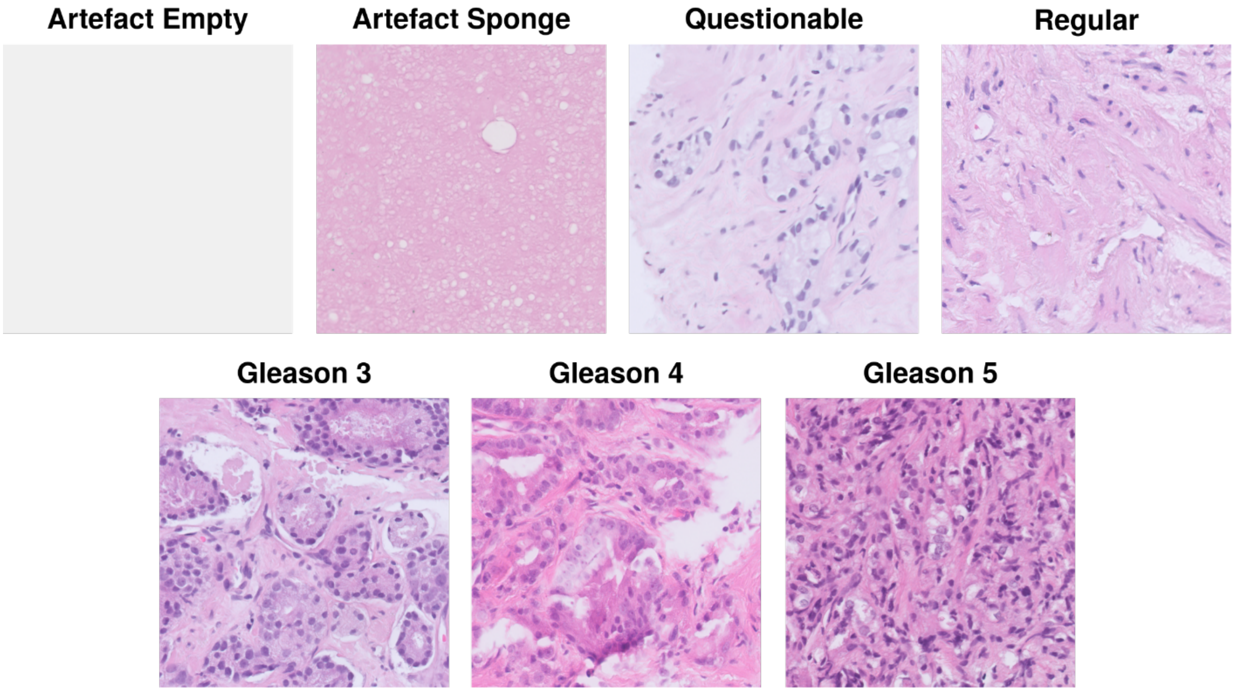

Figure S1. Visualization of tile classification from prostate carcinoma histological sections by the deployed deep neural network (AI) model.

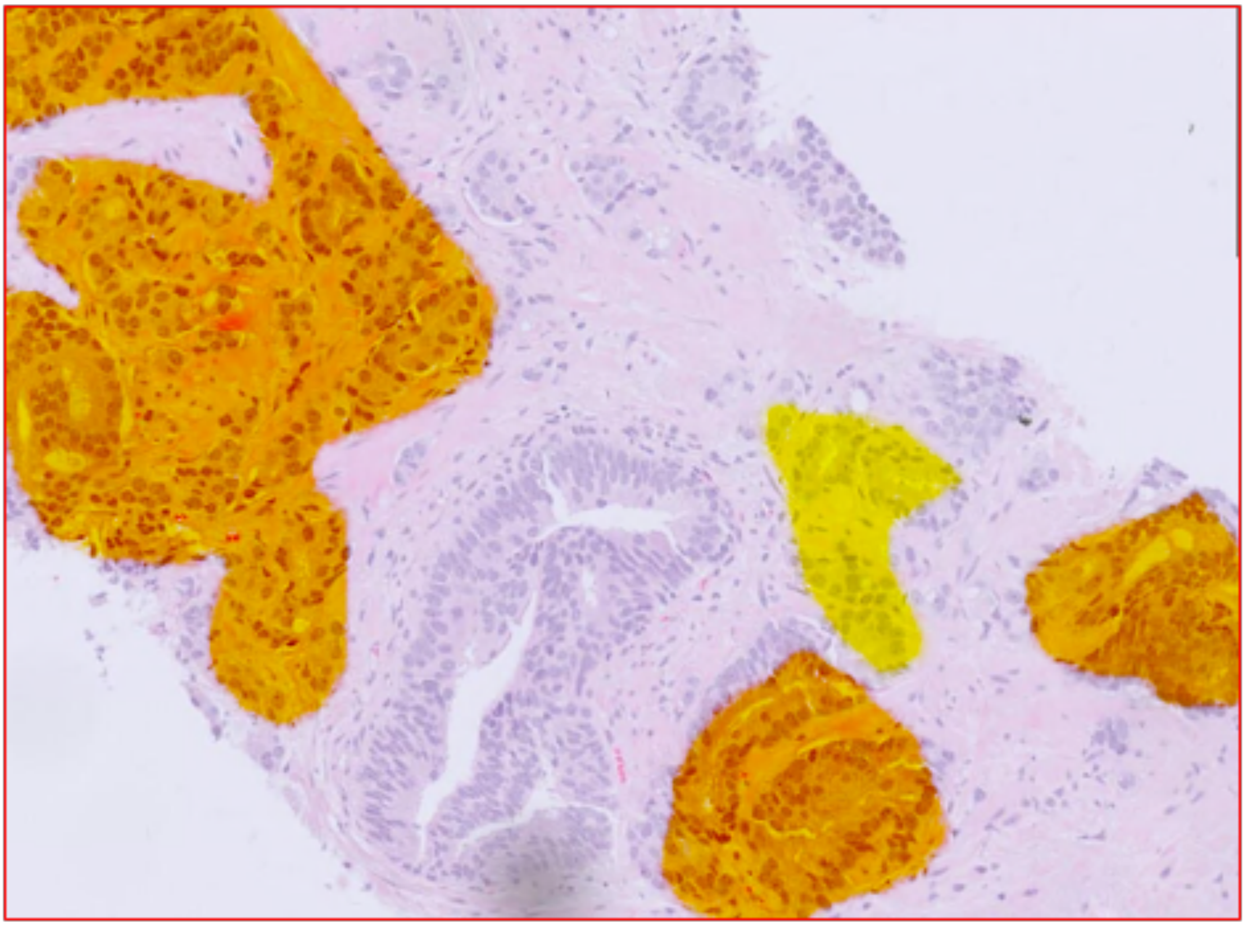

Figure S2. Annotations form a pathologist at the training dataset for Gleason grading.

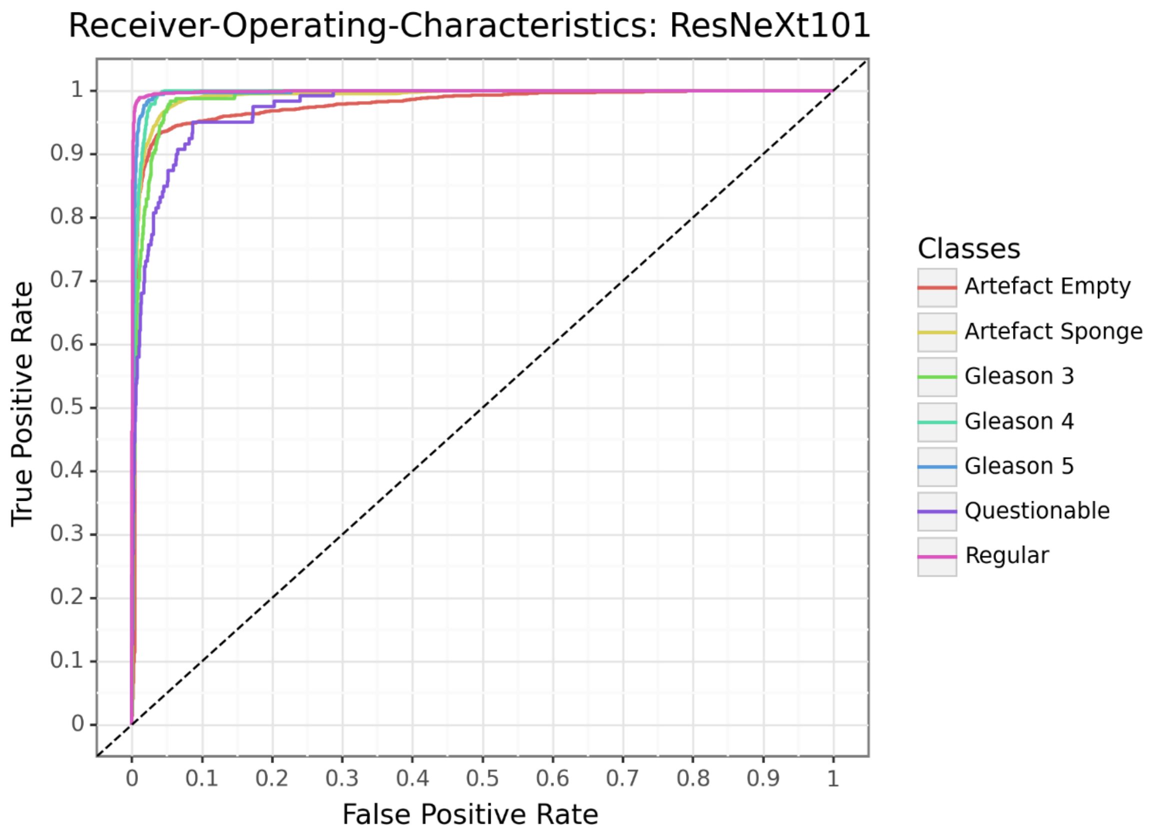

Figure S3. Area under the receiver operating characteristic curve (AUROC) of the AI pipeline.

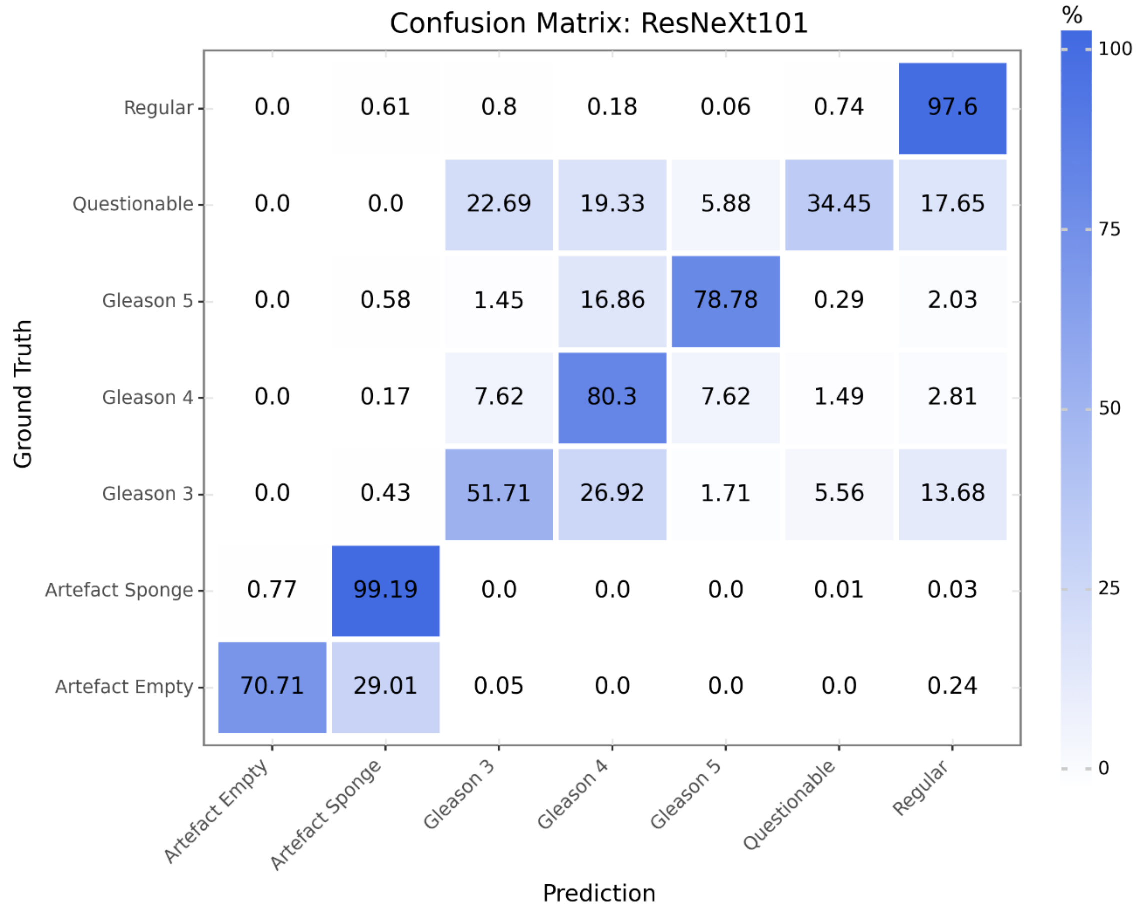

Figure S4. Confusion matrix of the AI pipeline.

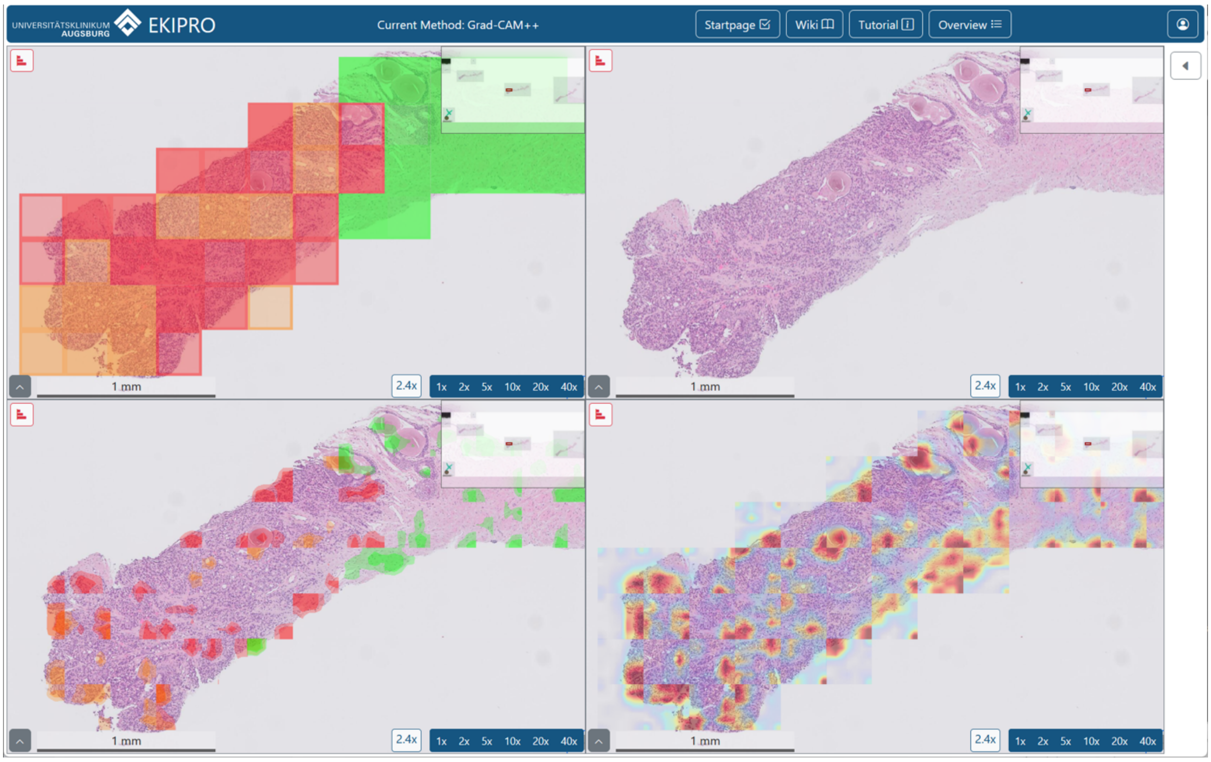

(A) Grad-CAM++

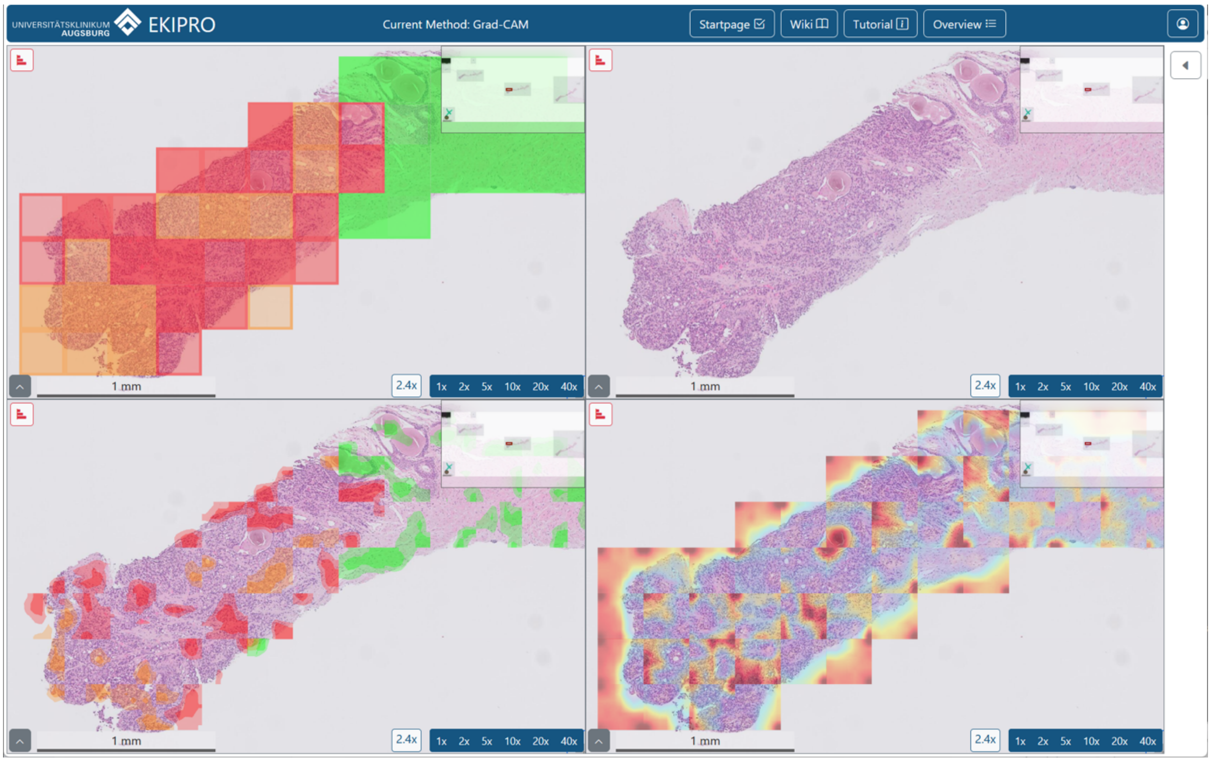

(B) Grad-CAM

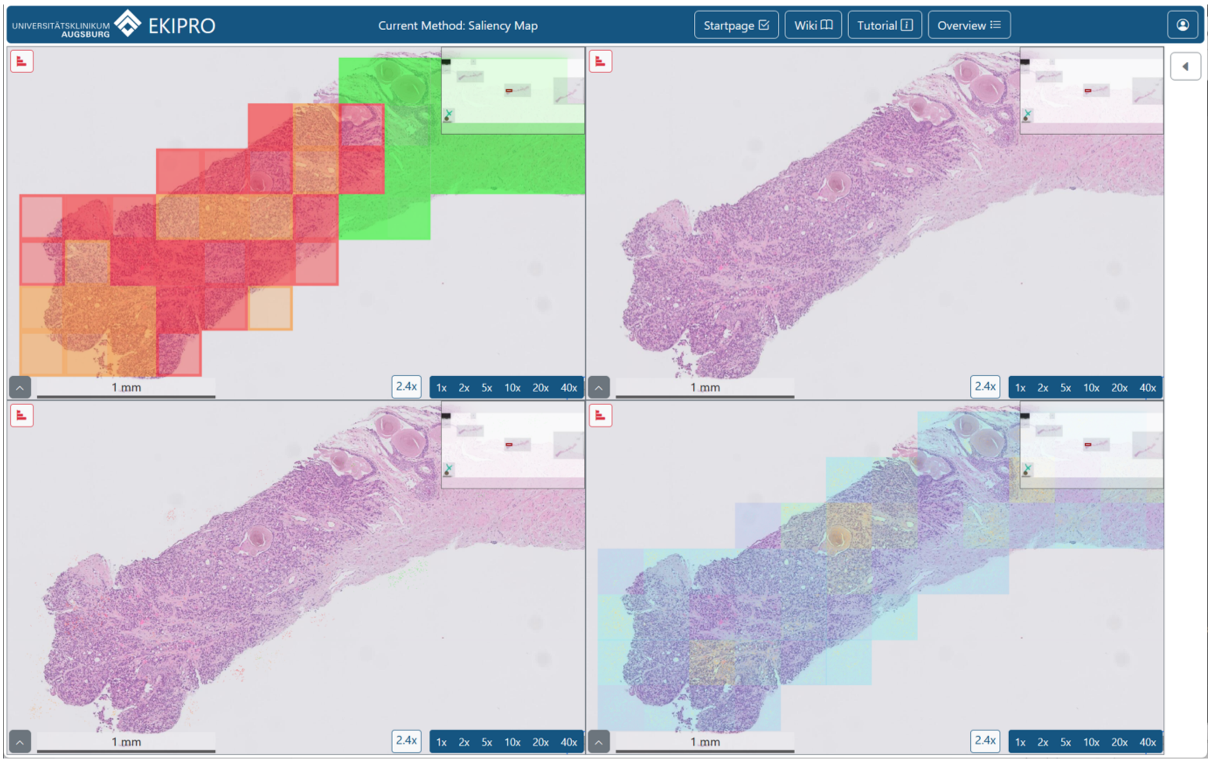

(C) Saliency Maps

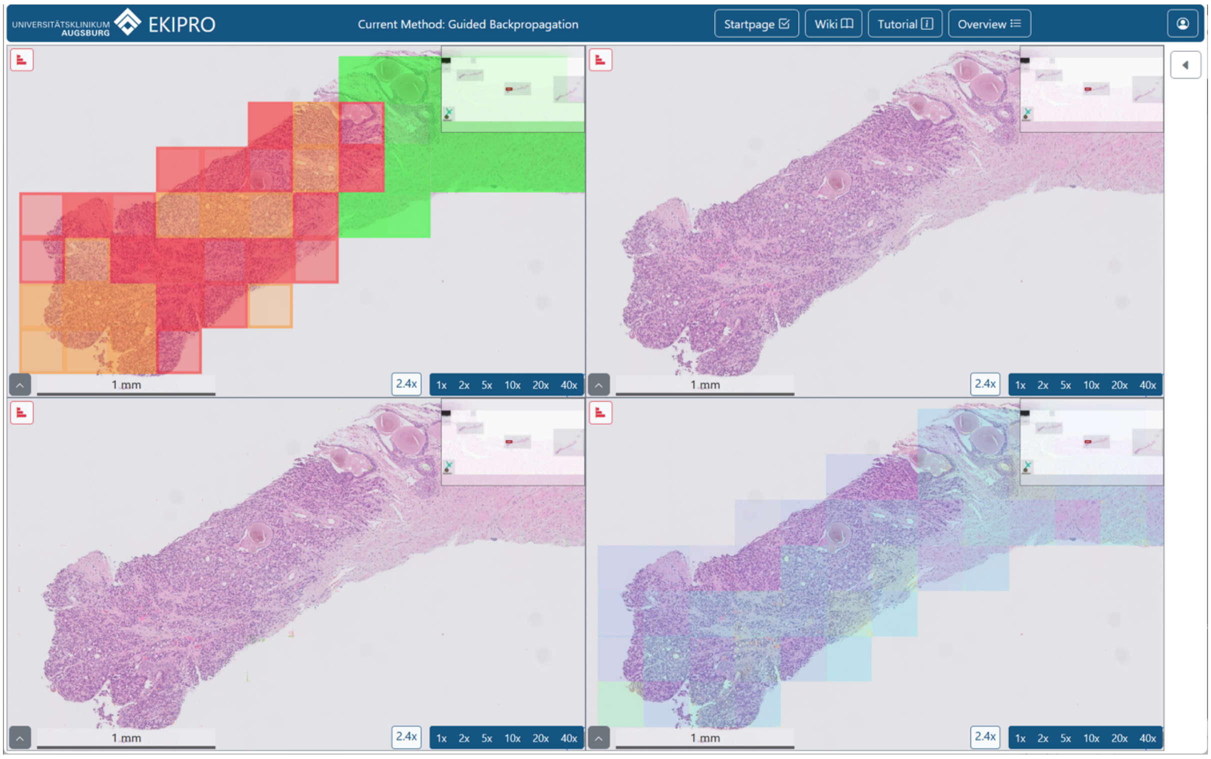

(D) Guided Backpropagation

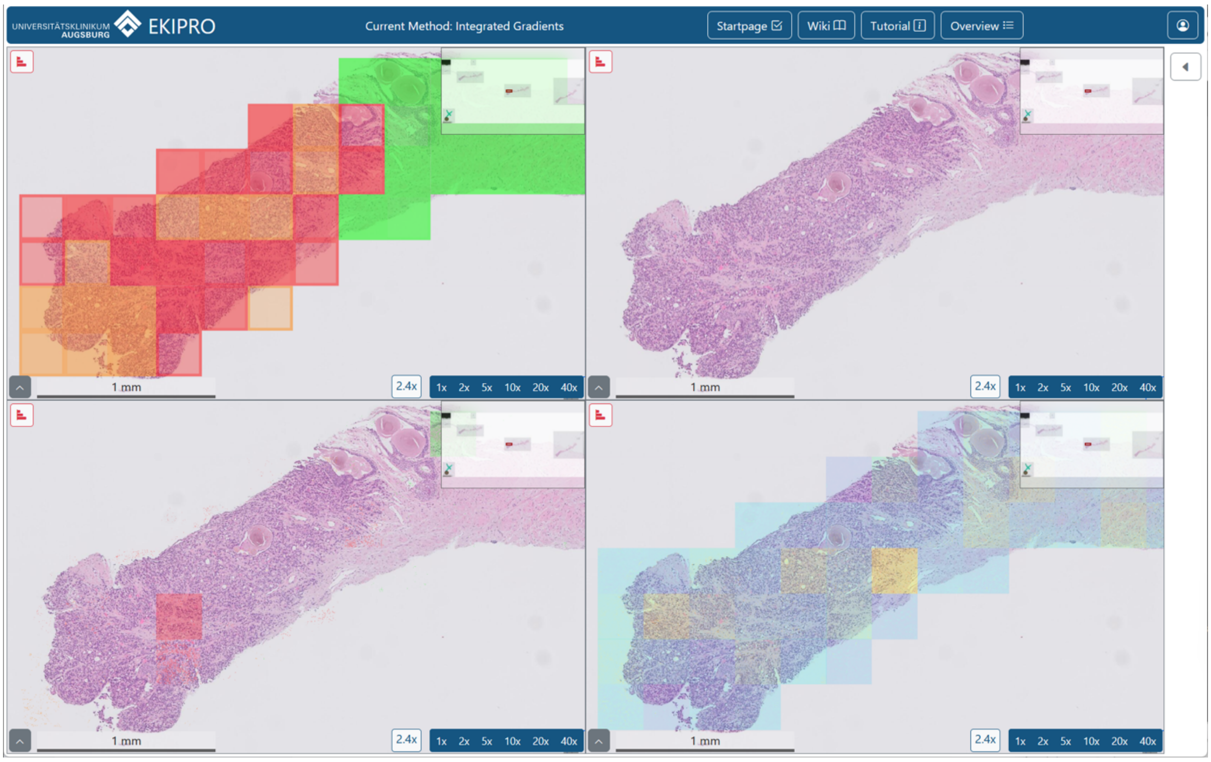

(E) Integrated Gradients

Figure S5. Screenshots taken from the EKIPRO evaluation tool, one for each XAI method (subfigures A–E) evaluated by the test group. In each quadrant, the same area of a punching biopsy is shown at 2.4× magnification. The upper right quadrant shows the sliced and H&E-stained biopsy without any overlays, while the upper left shows the tile-wise (1024 × 1024 pixels per tile) Gleason grade predictions by our AI model. For each XAI method, the lower left quadrant shows the tile-wise 'class-based' XAI visualizations while the lower right quadrant shows the tile-wise 'general attention' visualizations, as described in the AI model and XAI section of the manuscript.
For the prediction of the Gleason grades (upper left quadrant), only the grade with the highest probability is visualized for each tile. Opaque colors indicate a high probability, while translucent colors indicate a low probability.
The Gleason grades are color-coded: green = regular tissue, yellow = Gleason 3, orange = 4, red = Gleason 5. The same color-code is applied for the "class-based" XAI visualizations (lower left quadrant).
The 'general attention' XAI visualizations (lower right quadrant) are color-coded according to the 'Jet' heatmap color scheme, a gradient ranging from blue (0 = no attention) over yellow (0.5 = medium attention) to dark red (1 = high attention). For better visibility of high-attention regions, a linear gradient was applied to the transparency values of the colors, resulting in increasing opacity for higher values.

Table S1. Preference of class-based overlay per XAI method

Variable	*N*	%
Class-Based Overlay		
GC++	31/44	70
GC	33/44	75
SM	24/44	55
IG	27/44	61
GB	22/44	50
