# Supplementary material for: Do explainable AI (XAI) methods improve the acceptance of AI in clinical practice? An evaluation of XAI methods on Gleason grading
Source: J Pathol Clin Res. 2025 Mar 13;11(2):e70023. doi: 10.1002/2056-4538.70023 (PMC11904816; doi:10.1002/2056-4538.70023)
Supplement: Supplementary file 2 — File S1. User demographic questionnaire [file CJP2-11-e70023-s002.pdf]

Do explainable AI (XAI) methods improve the acceptance of AI in clinical practice? An evaluation of XAI methods on Gleason grading

R Manz *et al.*, *J Pathol Clin Res*, https://doi.org/10.1002/2056-4538.70023

File S1. User demographic questionnaire

In this file, you will find the introductory text and questions that users complete the first time they log in into the evaluation tool.

User Information

In this form we collect some additional information about our participants to enhance the outcomes of the evaluation.

Please note, that some Fields are required and marked with an asterix (*).

What range is your age?*

- <25
- 26
- 36–45
- 46–55
- >55

How many years of experience do you have as a pathologist?*

Do you have experience with the Gleason Score?*

- I do have experience
- I do not have experience

How would you rate your knowledge of the Gleason Score?

- 5 – A lot of experience
- 4 – Good experience
- 3 – Moderate experience
- 2 – Low experience
- 1 – Very Low experience

How frequently do you examine prostate Tumor samples in your everyday work?*

- Daily
- Weekly
- Monthly
- Yearly
- Never

Do you have experience with AI systems in pathology?*

- I do have experience
- I do not have experience

How would you rate your knowledge of AI systems in pathology?

- 5 – A lot of experience
- 4 – Good experience
- 3 – Moderate experience
- 2 – Low experience
- 1 – Very Low experience

How much interest do you have in AI applied to pathology?

- I have no interest in AI
- I have some interest – e.g., I read some articles when suggested
- I am moderately interested – e.g., I look out for articles regarding AI in pathology
- I am interested – e.g., I keep up to date with the current research in regards to AI in pathology
- I am very interested – e.g., I work actively with AI in pathology

Have you heard about eXplainable AI (XAI)?*

- Yes
- No
