# Supplementary material for: Do explainable AI (XAI) methods improve the acceptance of AI in clinical practice? An evaluation of XAI methods on Gleason grading
Source: J Pathol Clin Res. 2025 Mar 13;11(2):e70023. doi: 10.1002/2056-4538.70023 (PMC11904816; doi:10.1002/2056-4538.70023)
Supplement: Supplementary file 3 — File S2. User testing and evaluation questionnaires [file CJP2-11-e70023-s004.pdf]

Do explainable AI (XAI) methods improve the acceptance of AI in clinical practice? An evaluation of XAI methods on Gleason grading

R Manz *et al., J Pathol Clin Res,* https://doi.org/10.1002/2056-4538.70023

File S2. User testing and evaluation questionnaires

In this file, you will find the introductory text and questionnaires that users could read and complete for the evaluation. The same text is also displayed when starting an evaluation through the evaluation tool, https://ekipro.idm.uk-augsburg.science/en/viewer/. It begins with a brief explanation of the main components shown under 'Evaluation' and then continues with the evaluation questions.

Evaluation (see https://ekipro.idm.uk-augsburg.science/en/viewer/)

- The AI method classifies the sample, whereas the XAI methods mark areas that were relevant to the AI's decision, therefore the XAI provides the explanation for the AI classification.
- The upper left panel visualizes the Gleason Scoring AI classification.
- The upper right panel visualizes the plain sample.
- The lower left panel visualizes the selected XAI method using a Gleason Score heatmap. Thus, the heatmap of the XAI method is visualized for each class individually class.
- The lower right field visualizes the selected XAI method using a Jet heatmap. That means the heatmap is created across all classes.
- (more on the tutorial/wiki page)

[Questionnaire 1]

Grad-CAM:

The areas marked by the XAI method correspond with the regions of interest for determination of the Gleason Score within the prostate sample.

Strongly agree	Agree	Equal	Disagree	Strongly disagree
O	O	O	O	O

The XAI method marks irrelevant areas as relevant.

Strongly agree	Agree	Equal	Disagree	Strongly disagree
O	O	O	O	O

The XAI method highlights the relevant areas precisely.

Strongly agree	Agree	Equal	Disagree	Strongly disagree
O	O	O	O	O

The explanation of the different XAI methods is transparent and understandable.

Strongly agree	Agree	Equal	Disagree	Strongly disagree
O	O	O	O	O

Which visualization of the XAI method is best?

Classification based (Bottom left)	General (Bottom right)
O	O

Please write a short note on what you like/disliked about the current XAI method explanation.

Guided Backpropagation:

The areas marked by the XAI method correspond with the regions of interest for determination of the Gleason Score within the prostate sample.

Strongly agree	Agree	Equal	Disagree	Strongly disagree
O	O	O	O	O

The XAI method marks irrelevant areas as relevant.

○ Strongly agree ○ Agree ○ Equal ○ Disagree ○ Strongly disagree

The XAI method highlights the relevant areas precisely.

○ Strongly agree ○ Agree ○ Equal ○ Disagree ○ Strongly disagree

The explanation of the different XAI methods is transparent and understandable.

○ Strongly agree ○ Agree ○ Equal ○ Disagree ○ Strongly disagree

Which visualization of the XAI method is best?

○ Classification based (Bottom left) ○ General (Bottom right)

Please write a short note on what you like/disliked about the current XAI method explanation.

Saliency Map:

The areas marked by the XAI method correspond with the regions of interest for determination of the Gleason Score within the prostate sample.

○ Strongly agree ○ Agree ○ Equal ○ Disagree ○ Strongly disagree

The XAI method marks irrelevant areas as relevant.

○ Strongly agree ○ Agree ○ Equal ○ Disagree ○ Strongly disagree

The XAI method highlights the relevant areas precisely.

○ Strongly agree ○ Agree ○ Equal ○ Disagree ○ Strongly disagree

The explanation of the different XAI methods is transparent and understandable.

○ Strongly agree ○ Agree ○ Equal ○ Disagree ○ Strongly disagree

Which visualization of the XAI method is best?

○ Classification based (Bottom left) ○ General (Bottom right)

Please write a short note on what you like/disliked about the current XAI method explanation

Integrated Gradients:

The areas marked by the XAI method correspond with the regions of interest for determination of the Gleason Score within the prostate sample.

○ Strongly agree ○ Agree ○ Equal ○ Disagree ○ Strongly disagree

The XAI method marks irrelevant areas as relevant.

○ Strongly agree ○ Agree ○ Equal ○ Disagree ○ Strongly disagree

The XAI method highlights the relevant areas precisely.

○ Strongly agree ○ Agree ○ Equal ○ Disagree ○ Strongly disagree

The explanation of the different XAI methods is transparent and understandable.

- ○ Strongly agree
- ○ Agree
- ○ Equal
- ○ Disagree
- ○ Strongly disagree

Which visualization of the XAI method is best?

- ○ Classification based (Bottom left)
- ○ General (Bottom right)

Please write a short note on what you like/disliked about the current XAI method explanation.

Grad-CAM++:

The areas marked by the XAI method correspond with the regions of interest for determination of the Gleason Score within the prostate sample.

- ○ Strongly agree
- ○ Agree
- ○ Equal
- ○ Disagree
- ○ Strongly disagree

The XAI method marks irrelevant areas as relevant.

- ○ Strongly agree
- ○ Agree
- ○ Equal
- ○ Disagree
- ○ Strongly disagree

The XAI method highlights the relevant areas precisely.

- ○ Strongly agree
- ○ Agree
- ○ Equal
- ○ Disagree
- ○ Strongly disagree

The explanation of the different XAI methods is transparent and understandable.

Strongly agree	Agree	Equal	Disagree	Strongly disagree
○	○	○	○	○

Which visualization of the XAI method is best?

Classification based (Bottom left)	General (Bottom right)
○	○

Please write a short note on what you like/disliked about the current XAI method explanation.

[Ranking]

XAI-Priorization:

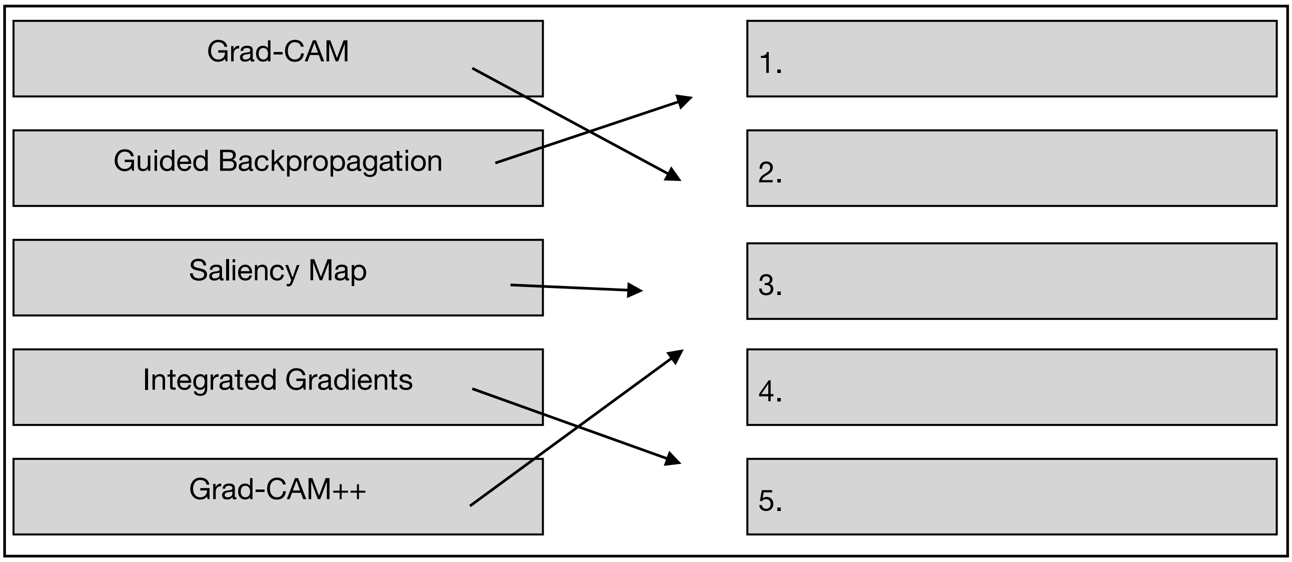

Which of the XAI methods helps best to explain the decision? Please create a ranking by dragging the labels from left to right. The one on top should be your favorite.

[Questionnaire 2]

General Evaluation Evaluation:

For this section you might want to check out the different XAI presets.

With the buttons below, you are able to set them again, without the need to open the specific evaluation sections

Grad-CAM	Saliency Map
Guided Backpropagation	Grad-CAM++
Integrated Gradients	General

The AI classifies the Gleason Score of the prostate sample correctly.

O	O	O	O	O
Strongly agree	Agree	Equal	Disagree	Strongly disagree

The Gleason Score is generally classified too high.

O	O	O	O	O
Strongly agree	Agree	Equal	Disagree	Strongly disagree

The Gleason Score is generally classified too low.

O	O	O	O	O
Strongly agree	Agree	Equal	Disagree	Strongly disagree

The AI classification simplifies the evaluation process of the prostate sample.

O	O	O	O	O
Strongly agree	Agree	Equal	Disagree	Strongly disagree

The use of AI leads to a more consistent grading of prostate carcinoma by different medical practitioner.

Strongly agree	Agree	Equal	Disagree	Strongly disagree
O	O	O	O	O

The different XAI methods support the comprehensibility of the AI results.

Strongly agree	Agree	Equal	Disagree	Strongly disagree
O	O	O	O	O

The XAI methods support the trustworthiness of the AI classification.

Strongly agree	Agree	Equal	Disagree	Strongly disagree
O	O	O	O	O

The explanations of the XAI methods were generally helpful.

Strongly agree	Agree	Equal	Disagree	Strongly disagree
O	O	O	O	O
