# Supplementary material for: Do explainable AI (XAI) methods improve the acceptance of AI in clinical practice? An evaluation of XAI methods on Gleason grading
Source: J Pathol Clin Res. 2025 Mar 13;11(2):e70023. doi: 10.1002/2056-4538.70023 (PMC11904816; doi:10.1002/2056-4538.70023)
Supplement: Supplementary file 4 — File S3. Tester feedback on the evaluated XAI methods [file CJP2-11-e70023-s003.pdf]

Do explainable AI (XAI) methods improve the acceptance of AI in clinical practice? An evaluation of XAI methods on Gleason grading

R Manz *et al.*, *J Pathol Clin Res*, https://doi.org/10.1002/2056-4538.70023

File S3. Tester feedback on the evaluated XAI methods (in German with English translation below):

Saliency Maps:

- Viele Areale ohne Gewebe werden als relevant markiert-Normalgewebe (Muskulatur) als Gleason 5 markiert (-)
- Hier kann ich die Erklärungen nicht wirklich nachvollziehen. Oft sind Bereiche ohne Gewebe besonders wichtig für die Entscheidung. (-)
- "Irritierend ist, dass die Markierungen sehr häufig in Regionen liegt, in denen Überhaupt kein Gewebe zu finden ist. (-)
- "Nichts"
- Klassenbasierte Version ist etwas anschaulicher in den wenigen Kacheln, in denen sie Überhaupt etwas markiert. Dann gibt es wieder große Abschnitte mit flächiger Färbung oder fehlender Darstellung. Allgemein wenig eingängliche Methode wegen der vielen kleinen Punkte. (- , + dropout)

GradCAM

- Weniger präzise als Grad CAM++ -größere Anfälligkeit für Artefakte (-)
- Diese Methode scheint größere Bereiche als wichtig fÜr die Entscheidung zu betrachten, als GRADCAM++. Ich finde die Kombination beider Visualisierungen gut. (+/-, + kombie aus beiden)
- Heatmap unten rechts ist visuell anschaulich, um Fokus der AI zu erkennen. (+, + JET)
- Obwohl alles gleaseon 4 tlw. unterschiedliche scores zwischen nebeinanderliegenden tiles. (-)
- Obwohl Läsion sehr homogen, tlw. unterschiedliche Gewichtung/Fokus der XAI (-)
- hier ist interessant (+)
- hier ist mir aufgefallen, dass die KI eine Perineuralscheideninfiltration nicht erkennt und als Normalgewebe markiert. (-)
- Insgesamt sind beide Darstellungen etwas schwierig zu vergleichen, da gefühlt sehr verschiedene Darstellung. (-/+)
- Rechts sehr viele leere Bereiche als für die KI relevant markiert. (- jet)
- XAI völlig unklar (-)
- Klassenbassierte Version ist gut nachvollziehbar (+, + dropout)
- gut nachvollziehbar. (+)
- Klassenbasierte Visualisierung ist deutlich verständlicher als die Allgemeine, die erscheint nicht präzise zu sein (+, + dropout)
- insgesamt korreliert die klassenbasierte Darstellung besser mit den interessanten Bereichen. Die Heatmap hebt teilweise nur die irrelevanten Bereiche hervor (+, + dropout, - jet)
- hier Markierungen teilweise nicht so präzise, da sehr flächige Tumoranteile, die eine ganze Kachel einnehmen (-)

GradCAM++

- nur einzelne Artefakte als relevant markiert (-)
- Unten rechts allgemeine Heatmap gut. (+, +jet)
- Pro: erkennt Areale die wichtig sind richtig Con: einige wichtige Areale aber nicht erkannt Ergebnis insgesamt trotzdem gut (+)
- die Darstellung rechts unten empfinde ich persönlich als höchst verwirrend. Wenn man zoomt, ist es sehr gut verständlich, aber in der Übersicht will ich aufgrund der dunkelroten Farbe intuitiv annehmen, dass das Gewebe (hoch)maligne ist, obwohl ich verstanden habe, dass nur die Relevanz des Gewebes markiert wird. (- jet)
- "Im Vergleich zu anderen Methoden schön präzise Markierung. (+)
- Es ist schnell und einfach zu visualisieren. (+)
- Unklare bzw. limitierte Bereiche (-)
- sehr gut nachvollziehbare Darstellung und hilfreich (+)
- die markierten Bereiche sind Überwiegend auch pathologisch relevant, es fehlen aber in den meisten Kacheln auch relevante Abschnitte. Beide Visualisierungen zeigen das gleiche, mir gefällt persönlich die Klassenbasierte Darstellung etwas besser (+/-, + dropout)

Integrated Gradients

- Pixelbasiert einfach schwierig zu interpretieren für mich persönlich (generelles finding für alle) (-)
- ich kann rechts unten leider gar nichts erkennen (nur eine normale HE?) (- jet)
- Teilweise sehr kleine Markierungen, die kaum auffindbar sind. (-)
- Allgemein fehlt, Klassenbasiert zu wenig Auflösung (-)
- Zu wenig markierte Bereiche (-)
- Ähnliches Problem wie GB und SM (-)

Guided Backpropagation

- Ich konnte die Erklärungen der KI nicht wirklich nachvollziehen. Ihre Entscheidungen waren aber ziemlich zutreffend. (-)
- Finde sehr interessant (+)
- auch hier werden glaube ich relevante Bereiche nicht erkannt (-)
- Für mich eine nicht intuitive Darstellung. (-)
- Pixel schwierig zu finden und meist irrelevant"" (-)
- in der Stufe mit grüner Farbmarkierung (auf Objektträger) ist m.E. der Gleason (3) zu niedrig. Ich denke hier eher an Gleason 5, evtl. Vakuolen im Zytoplasma als Drüsenlumen erkannt. (?)
- pixel basierte Darstellung in zweiter Näherung gut nachvollziehbar (+)
- In einigen Bereichen markiert die Methode zwar relativ gut die Tumorzellen, in anderen Kacheln fehlt dann aber wieder ein nachvollziehbarer Zusammenhang, oder die Markierungen sind zu schwach. In der Klassenbasierten Auswertung gibt es kacheln, die (+/-)

Tester feedback on the evaluated XAI methods (translated from German into English with deepL.com – free version):

Saliency maps:
- Many areas without tissue are marked as relevant-normal tissue (muscle) is marked as Gleason 5 (-)
- I can't really understand the explanations here. Areas without tissue are often particularly important for the decision. (-)
- "It is irritating that the markings are very often located in regions where no tissue is found at all. (-)
- "Nothing"
- Class-based version is slightly more descriptive in the few tiles where it marks anything at all. Then there are again large sections with flat coloring or no representation. Generally not a very catchy method because of the many small dots. (- , + dropout)

GradCAM
- Less precise than Grad CAM++ - greater susceptibility to artifacts (-)
- This method seems to consider larger areas as important for the decision than GRADCAM++. I like the combination of both visualizations. (+/-, + combination of both)
- Heatmap bottom right is visually descriptive to recognize focus of AI. (+, + JET)
- Although everything gleaseon 4 partly different scores between adjacent tiles. (-)
- Although lesion very homogeneous, partly different weighting/focus of the XAI (-)
- here is interesting (+)
- I noticed that the AI does not recognize a perineural sheath infiltration and marks it as normal tissue. (-)
- Overall, both images are somewhat difficult to compare, as they appear very different. (-/+)
- On the right, many empty areas marked as relevant for AI. (- jet)
- XAI completely unclear (-)
- Class-based version is easy to understand (+, + dropout)
- well comprehensible. (+)
- Class-based visualization is much easier to understand than the general one, which does not appear to be precise (+, + dropout)
- Overall, the class-based visualization correlates better with the areas of interest. The heatmap sometimes only highlights the irrelevant areas (+, + dropout, - jet)
- markings here are sometimes not as precise, as very flat tumor parts that take up an entire tile (-)

GradeCAM++
- only individual artifacts marked as relevant (-)
- Bottom right general heatmap good. (+, +jet)
- Pro: recognizes areas that are important correctly Con: some important areas but not recognized Overall result still good (+)

- I personally find the display at the bottom right highly confusing. If you zoom in, it is very easy to understand, but in the overview, the dark red color makes me intuitively assume that the tissue is (highly) malignant, although I understood that only the relevance of the tissue is marked. (- jet)
- "Nicely precise marking compared to other methods. (+)
- It is quick and easy to visualize. (+)
- Unclear or limited areas (-)
- Very easy to understand and helpful (+)
- the marked areas are also predominantly pathologically relevant, but relevant sections are also missing in most tiles. Both visualizations show the same thing, I personally like the class-based representation a little better (+/-, + dropout)

Integrated Gradients

- Pixel-based simply difficult to interpret for me personally (general finding for all) (-)
- I can't see anything at the bottom right (just a normal HE?) (- jet)
- Some of the markings are very small and hard to find. (-)
- Generally missing, class-based too little resolution (-)
- Too few marked areas (-)
- Similar problem as GB and SM (-)

Guided backpropagation

- I couldn't really understand the AI's explanations. But their decisions were pretty accurate. (-)
- Find very interesting (+)
- I think relevant areas are not recognized here either (-)
- For me an unintuitive presentation. (-)
- Pixel difficult to find and mostly irrelevant"" (-)
- in the stage with green color marking (on slide), the Gleason (3) is too low in my opinion. I rather think of Gleason 5, possibly vacuoles in the cytoplasm recognized as glandular lumen. (?)
- pixel-based representation in second approximation well comprehensible (+)
- In some areas, the method marks the tumor cells relatively well, but in other tiles there is no comprehensible connection, or the markings are too weak. In the class-based evaluation, there are tiles that (+/-)
